# Toward an integrated animal–human nutrition intelligence via agentic retrieval-augmented language models

**DOI:** 10.3389/fnut.2026.1815038

**Published:** 2026-09-10

**Authors:** Luis O. Tedeschi, Nicole Greer, Karun Kaniyamattam, Dheeraj Mudireddy, Robert Strong, Praneet Sai Madhu Surabhi, Jian Tao, Ashley Wang

**Affiliations:** 1 Department of Animal Science, Texas A&M University, College Station, TX, United States; 2 Department of Computer Science, Texas A&M Institute of Data Science, Texas A&M University, College Station, TX, United States; 3 Department of Agricultural Leadership, Education & Communications, Texas A&M University, College Station, TX, United States; 4 College of Performance, Visualization & Fine Arts, Department of Electrical & Computer Engineering, Texas A&M Institute of Data Science, Texas A&M University, College Station, TX, United States; 5 School of Public Health, Texas A&M University, College Station, TX, United States

**Keywords:** animal nutrition, animal-source foods, decision support systems, evidence-grounded synthesis, human nutrition, large language models, literature synthesis, retrieval-augmented generation

## Abstract

Animal-source foods are nutritionally “plastic,” with fatty acid profiles, vitamins, and minerals that can be altered by animal diets and management. Although the literature documenting these effects is extensive, it is fragmented across disciplines and experimental contexts, limiting translation into actionable guidance for food quality and human nutrition. We developed the Intelligent System for Integrating Global Human & Animal Health Technology (INSIGHT), a domain-specialized retrieval-augmented generation (RAG) system designed to synthesize evidence across animal production and human nutrition research with explicit provenance. INSIGHT employs a nine-stage RAG pipeline that integrates query expansion, hybrid retrieval, evidence reranking, and self-verification to deliver transparent, citation-linked responses. Throughout, retrieval refers to document selection, integration to the combination of retrieved evidence into a coherent evidence set, and synthesis to the LLM-based generation of grounded narrative responses. The knowledge base comprises ~4,000 peer-reviewed papers in animal science, feed composition, and human dietary research. To evaluate retrieval performance across diverse literature contexts, we developed a multi-group evaluation framework: 282 documents were randomly selected and organized into 26 semantically coherent groups of ~10 papers each. For each group, Perplexity Deep Research generated 25 question–answer pairs and identified ground-truth relevant documents. Each question was posed to INSIGHT, yielding document-level precision, recall, and F1-score metrics across 614 total queries. Generalized linear mixed models with beta regression revealed significant between-group performance variation (*p* < 0.0001), indicating that retrieval effectiveness depends on semantic domain characteristics. Across groups, INSIGHT achieved a mean precision of 0.77 (SD = 0.12), recall of 0.62 (SD = 0.15), and F1-score of 0.67 (SD = 0.10), all significantly exceeding a 0.5 baseline (*p* < 0.0001). These results demonstrate that INSIGHT provides reliable document-level retrieval across diverse topical domains, though significant between-group performance variation (*p* < 0.0001) indicates that retrieval effectiveness is context-dependent. The present evaluation is intentionally scoped as a controlled retrieval performance assessment; end-to-end synthesis quality evaluation using systematic review benchmarking and RAG assessment metrics represents a critical next step. Domain-specialized, evidence-grounded systems such as INSIGHT can accelerate cross-disciplinary knowledge integration, with continued development focused on improving recall in semantically complex domains, enhancing terminology normalization, and expanding corpus coverage across animal production and human nutrition research.

## Introduction

Animal-source foods, such as milk, meat, and eggs, are biochemically plastic as their nutritional profiles (e.g., fatty acids, vitamins, and minerals) may change depending on what animals eat and, in some circumstances, how they are managed (1). When consumed by humans, these nutrient-dense foods contribute bioavailable macronutrients and micronutrients that support growth, development, and health across the life course (2).^1^

Beef and dairy products illustrate how feeding strategies can meaningfully alter the lipid profile of animal-source foods. In beef cattle, grass-based diets consistently increase omega-3 fatty acids, conjugated linoleic acid (CLA), and antioxidant content relative to grain-finishing systems, though sensory attributes such as flavor and tenderness may vary depending on production context (3–5). In dairy cows, dietary composition and rumen biohydrogenation pathways are key determinants of milk fat quality. Supplementation with specific fat sources, such as flaxseed, linseed, or fish oil, enhances the proportion of omega-3 fatty acids in milk, while targeted manipulation of rumen biohydrogenation can increase CLA content (6–9). These dietary interventions not only improve the nutritional value of animal-source food by elevating bioactive fatty acids but also enhance antioxidant capacity, contributing to product stability. Importantly, human studies link higher intakes of omega-3 fatty acids and CLA with reduced cardiovascular disease risk and potential anti-inflammatory benefits, underscoring the public health relevance of feed-driven modifications in the composition of animal-source foods (10). Thus, manipulating cow diets can produce “healthier” milk and beef profiles relevant to public health nutrition. However, it is essential to note that specific CLA isomers, particularly trans-10,cis-12 CLA, can induce milk fat depression (MFD) in dairy cows. Research has demonstrated that this particular CLA isomer decreases milk fat synthesis in a dose-dependent manner through anti-lipogenic effects, reducing the mRNA abundance of key lipogenic genes involved in mammary fat synthesis (11).

Beyond fats, other dietary interventions can also elevate the nutritional quality of animal-source foods. The vitamin D status of dairy cows, which is affected by sunlight exposure or dietary supplementation, changes the vitamin D content of their milk. Seasonal variation is evident, with higher levels in summer and lower levels in winter, unless compensated for by supplements (12, 13). Improving cow vitamin D status through feeding or fortification therefore has direct public health implications given the widespread association of deficiency with rickets, osteoporosis, and immune dysfunction (14). Similarly, supplementing dairy cows with vitamin E enhances the oxidative stability of milk, lowers somatic cell counts (SCC), and reduces the risk of mastitis, thereby improving overall milk quality (15).

Dietary selenium (Se) supplementation plays a dual role in dairy production, enhancing cow health and enriching animal products with bioavailable Se, a cofactor of glutathione peroxidase (GSH-Px). Supplementation increases plasma GSH-Px activity, reduces SCC, and increases milk Se concentrations, improving oxidative stability (16–18). For human consumers, Se-enriched dairy products are valuable functional foods, particularly in regions with Se-poor soils where dietary deficiency contributes to impaired immune function and thyroid disorders (19). Chromium (Cr), especially in organic forms, improves glucose metabolism and reduces stress responses in livestock. In dairy cows, Cr supplementation enhances feed intake, immune function, and antioxidant status, indirectly supporting milk yield and quality (20, 21). In beef cattle, Cr propionate supplementation improves growth efficiency and carcass characteristics, increasing lean tissue deposition and dressing percentage (22, 23). These effects stem from Cr′s role in enhancing insulin sensitivity and redirecting nutrients toward lean tissue, with economic and sensory benefits aligned with consumer demand for leaner meat profiles.

Importantly, the nutritional contribution of animal-source foods must be interpreted within the broader context of human dietary patterns, where protein sources differ substantially in amino acid composition, digestibility, and associated micronutrients. Animal- and plant-sourced proteins are not nutritionally interchangeable; plant proteins frequently exhibit limiting essential amino acids and lower digestibility, whereas animal-source proteins typically provide more complete amino acid profiles and higher bioavailability (24). This source-aware perspective underscores the importance of linking animal feeding strategies not only to food composition, but also to how animal-source foods function within mixed human diets.

Although the effects of feeding strategies on the nutritional composition of animal-source foods are well-documented, the evidence is dispersed across various journals, species, and experimental designs, making it challenging to synthesize and translate into practical guidance for human health. This fragmentation of agricultural knowledge has been recognized as a critical barrier to the development of agricultural artificial intelligence (AI) applications, with existing systems often lacking the contextual understanding necessary for effective decision support (25). Emerging resources such as the United States Department of Agriculture’s (USDA) FoodData Central^2^ now provide detailed composition data with metadata on sample source and production practices, offering opportunities to link feeding strategies with human-relevant nutrient profiles. Yet, fully realizing this potential requires advanced tools that can integrate heterogeneous literatures, ontologies, and databases.

The substantial time and resource burden of conventional systematic review methods compounds the challenge of synthesizing fragmented agricultural and nutrition literature. Empirical analyses of medical systematic reviews demonstrate that a single review requires an average of 67.3 weeks and a team of five researchers to complete (26), with estimated labor costs ranging from $141,000 to $180,000 per review, assuming a research effort of 15 h out of a 40-h workweek dedicated to the review and a base annual salary of $74,160 to $82,090 (27)—costs that are likely comparable for systematic reviews in animal science and nutrition given similar literature search and synthesis demands. As the volume of primary literature continues to grow exponentially, the cumulative cost of manually synthesizing evidence across the animal science, feed composition, and human nutrition domains represented in this work would reach tens of millions of dollars. This barrier effectively limits the translation of research findings into actionable guidance for producers, nutritionists, and policymakers. Intelligent systems capable of accelerating evidence synthesis at scale are therefore not merely a convenience but an economic and scientific necessity.

Recent dietary guidance further illustrates how rapidly public-facing nutrition frameworks evolve and how underlying assumptions about food sources shape interpretation. For example, recent U. S. dietary guidance^3^ has introduced a “Real Food Pyramid” that places animal-source foods—such as meat, dairy, eggs, and fish—alongside fruits and vegetables as foundational components of the diet, while de-emphasizing grains, legumes, and highly processed foods. This reframing contrasts with earlier dietary pyramids that emphasized cereal grains as a primary base and highlights ongoing shifts in how protein quality, nutrient density, and food processing are weighted in dietary recommendations. Such changes reinforce the need for transparent, evidence-traceable systems capable of linking dietary guidance to the underlying animal production practices, compositional data, and scientific literature on which these recommendations implicitly rely.

Recent advances in large language models (LLM), i.e., AI systems trained on vast textual corpora, have substantially improved machine performance on tasks central to scientific work, including literature synthesis, ethical reasoning, and replication of known empirical patterns. Systematic evaluations show that, while LLM remain imperfect, their capabilities are rapidly evolving, with newer generations exhibiting reduced hallucination rates and improved self-awareness of uncertainty (28). Nevertheless, hallucination remains a fundamental limitation: LLM may confidently generate incorrect or fabricated content, including fictitious citations and unverifiable claims. This behavior poses significant risks for scientific reliability and decision-making, particularly in high-stakes applications (29).

Emerging applications of LLM in nutrition have revealed both capabilities and fundamental constraints. Frontier models (Claude Opus 4.6, Gemini 3 Pro, GPT-5.1) achieved 90–94% agreement with human experts in dietary classification tasks, yet all pairwise model comparisons showed statistically significant differences (*p* < 0.001), and prompt structure alone altered classification outcomes substantially (30). Note that currently the latest versions are Claude Sonnet 5 and GPT-5.5. These findings illustrate that parametric knowledge, even in state-of-the-art models, produces inconsistent reasoning and remains fundamentally limited by the boundaries of the training data, a challenge that is amplified when extending from bounded classification tasks to cross-domain literature synthesis that requires integrating heterogeneous evidence sources.

To address hallucination and knowledge-boundary limitations, Retrieval-Augmented Generation (RAG) has emerged as an effective architectural strategy. By conditioning LLM outputs on retrieved external documents, RAG improves factual grounding, traceability, and temporal relevance. Empirical studies demonstrate that RAG-based systems significantly enhance accuracy and trustworthiness in knowledge-intensive tasks, even under imperfect retrieval conditions (31, 32). However, domain-specific analyses caution that LLM—even when augmented—remain fundamentally data-driven and lack embedded biological or mechanistic causality. Accordingly, their responsible deployment in applied sciences requires careful curation of retrieval sources, transparency, and sustained human oversight to ensure that AI systems complement rather than supplant mechanistic models and expert judgment (33).

The feasibility of RAG architectures for nutrition applications has been demonstrated in adjacent domains. Gavai and van Hillegersberg (34) developed an AI-driven dietary recommendation system that combines RAG with a locally deployed LLaMA3 model to generate personalized smoothie recipes for individuals with obesity and type 2 diabetes, integrating dietary guidelines from the Dutch National Institute for Public Health and the Environment, USDA FoodData Central, and the American Diabetes Association as its retrieval corpus. Evaluated across 1,000 generated recipes, the system achieved 80.1% adherence to nutritional guidelines and 92% compliance with sustainability criteria, illustrating that RAG-based systems can translate complex nutritional evidence into actionable, evidence-grounded outputs. Importantly, however, that system addresses a patient-facing guidance task (i.e., retrieving from established clinical guidelines to produce individualized dietary recommendations) rather than synthesizing primary scientific literature. The INtelligent System for Integrating Global human and animal Health Technology (INSIGHT) targets a complementary but distinct challenge: integrating heterogeneous research evidence across the animal science and human nutrition literature to support evidence synthesis at the upstream scientific level, where the guidelines that patient-facing systems rely upon are themselves constructed.

The broader landscape of domain-specialized LLM includes several systems relevant to contextualizing INSIGHT. In the biomedical domain, BioGPT (35) demonstrated that pretraining a generative transformer model exclusively on PubMed literature substantially improved performance on biomedical text mining tasks including relation extraction, question answering, and document classification relative to general-purpose models. Similarly, BioMedLM (36) showed that a 2.7 billion parameter model trained exclusively on PubMed abstracts and full-text articles could match or exceed much larger general-purpose models on biomedical question answering benchmarks, suggesting that corpus specificity rather than model scale is a primary driver of domain performance. In the clinical domain, GatorTron (37) demonstrated that training on 82 billion words of de-identified clinical notes from the University of Florida Health system produced a model that outperformed existing biomedical and clinical transformer models on five clinical Natural Language Processing (NLP) tasks, underscoring the value of domain-specific training data even when that data is institutionally constrained. At the level of scientific literature synthesis, Asai et al. (38) introduced OpenScholar, a retrieval-augmented system that retrieves from a corpus of 45 million open-access papers across computer science, biomedicine, physics, and neuroscience, demonstrating that a specialized 8B model with iterative self-feedback can outperform GPT-4o on multi-paper synthesis tasks and produce responses preferred over expert-written answers by domain scientists. In the nutrition domain specifically, Gavai and van Hillegersberg (34) demonstrated the feasibility of RAG-based systems for personalized dietary guidance, while Ase et al. (30) showed that frontier general-purpose LLM can achieve high agreement with human experts on bounded dietary classification tasks. However, none of these systems address the cross-domain challenge of synthesizing primary scientific literature across animal production practices and human nutrition outcomes, the specific gap INSIGHT is designed to fill.

The development of domain-specific LLM represents a well-established and empirically validated approach that consistently demonstrates superior performance compared to general-purpose models for specialized applications, providing strong scientific justification for a system’s domain-focused architecture. The most compelling evidence emerges from Bloomberg’s landmark BloombergGPT study, where Wu et al. (39) developed a 50-billion parameter model trained on 363 billion financial tokens combined with 345 billion general-purpose tokens, demonstrating that the specialized model “outperforms existing models on financial tasks by significant margins without sacrificing performance on general LLM benchmarks,” with particularly strong advantages in domain-specific document categories such as financial filings and industry-specific content. This pattern of domain superiority extends across multiple specialized fields, as evidenced by Xie et al. (40) in the medical domain, whose Me-LLaMA model “outperforms existing open medical LLM in zero-shot and supervised settings and surpasses ChatGPT and GPT-4 after task-specific instruction tuning for most text analysis tasks,” highlighting the critical importance of “combining domain-specific continual pretraining with instruction tuning to enhance performance.” The theoretical foundation for this approach is well-established, with Xie et al. (40) defining domain specialization as “customizing general-purpose LLM according to specific domain contextual data, augmented by domain-specific knowledge, optimized by the domain’s objective, and regulated by domain-specific constraints. Beyond performance advantages, domain-specific models offer significant practical benefits, as Kerner (41) demonstrated that “smaller, specialized LLM that perform well on tasks in a certain domain” can outperform larger general models while providing “comparatively fast inference times, lower latency, and less expensive training.” The most successful implementations employ mixed training strategies to maintain broad language capabilities while achieving domain excellence, providing a proven framework for the development of domain-specific system capable of bridging animal science and human nutrition research with superior accuracy and contextual understanding compared to general-purpose alternatives.

Building on these advances, we propose INSIGHT, a domain-tuned, agentic RAG model designed to read across animal science and human nutrition, link findings to composition databases, and generate transparent, evidence-grounded syntheses. Prior work has shown that while large pre-trained language models store considerable knowledge in their parameters, their performance often lags on knowledge-intensive tasks due to hallucinations and limited ability to update domain knowledge. The RAG approach mitigates these shortcomings by combining parametric memory with non-parametric retrieval, resulting in outputs that are more factual, specific, and diverse. In benchmark evaluations, RAG models consistently outperformed parametric-only LLM and task-specific extractive architectures on open-domain question-answering, abstractive generation, and fact verification tasks (42). However, the extent to which these general RAG performance advantages translate to highly specialized domains remains an open empirical question. Critical uncertainties include the minimum corpus size required for domain-specific advantage, the optimal balance between retrieval precision and recall for evidence synthesis, and whether document-level grounding translates to superior answer quality as perceived by domain experts. We hypothesize that a domain-adapted RAG system such as INSIGHT will provide more transparent and verifiable evidence grounding than a general-purpose LLM, though the trade-offs between retrieval coverage, answer completeness, and narrative synthesis quality in specialized nutrition domains require systematic evaluation. Accordingly, the present study is scoped as a proof-of-concept retrieval evaluation that establishes INSIGHT’s document identification capabilities as a necessary prerequisite for downstream synthesis quality assessment. End-to-end synthesis quality evaluation, including systematic review benchmarking and semantic similarity scoring, is identified as the primary direction for future work.

## Materials and methods

### INSIGHT system development

Throughout this manuscript, three related but distinct terms are used with specific technical meanings that reflect different stages of the INSIGHT pipeline. *Retrieval* refers specifically to the document-selection process encompassing Stages 2–7 of the RAG pipeline, in which relevant passages are identified, ranked, and selected from the corpus in response to a user query. *Integration* refers to the process of combining information from multiple retrieved sources into a coherent evidence set that collectively addresses the query. *Synthesis* refers to the LLM-based generation of a coherent narrative response grounded in the integrated evidence, corresponding to Stage 8 of the pipeline. These distinctions are consistently maintained throughout the manuscript to reflect the sequential, functionally distinct nature of each process within the INSIGHT architecture.

#### Design goals

The INSIGHT system was designed around three core principles to ensure reliable and comprehensive nutrition research synthesis. First, the system prioritizes truthful synthesis with provenance by citing source passages and displaying retrieval context to maintain transparency and verifiability. Second, it employs an agentic workflow that enables the model to autonomously decide when to search, which tools to utilize, and when to escalate findings to human reviewers for validation. Finally, the system emphasizes interoperability by bridging the language and concepts of both animal science and human nutrition through integrated ontologies and dataset adapters.

#### Model backbone and serving

The INSIGHT system uses an open-source LLM to enable cost-effective domain adaptation and transparent deployment. Specifically, the system employs LLaMA-3.1-8B (43) for answer verification and Gemma-7B (44) for query expansion and response generation. The LLaMA-3.1 model family, spanning 8B to 405B parameters, offers widely supported, well-documented options for enterprise use, facilitating reproducibility and scalability in research applications (43). Models were served locally using Ollama,^4^ enabling on-premises deployment without external API dependencies. The two models occupy distinct, non-competing roles: Gemma-7B performs query expansion (Stage 3) and evidence-grounded response generation (Stage 8), whereas LLaMA-3.1-8B performs independent self-verification (Stage 9). Using a model from a different family for verification is a deliberate safeguard against the correlated, self-confirming errors that can occur when a single model both generates and judges its own output.

#### Retrieval-augmented generation

**Figure 1:**
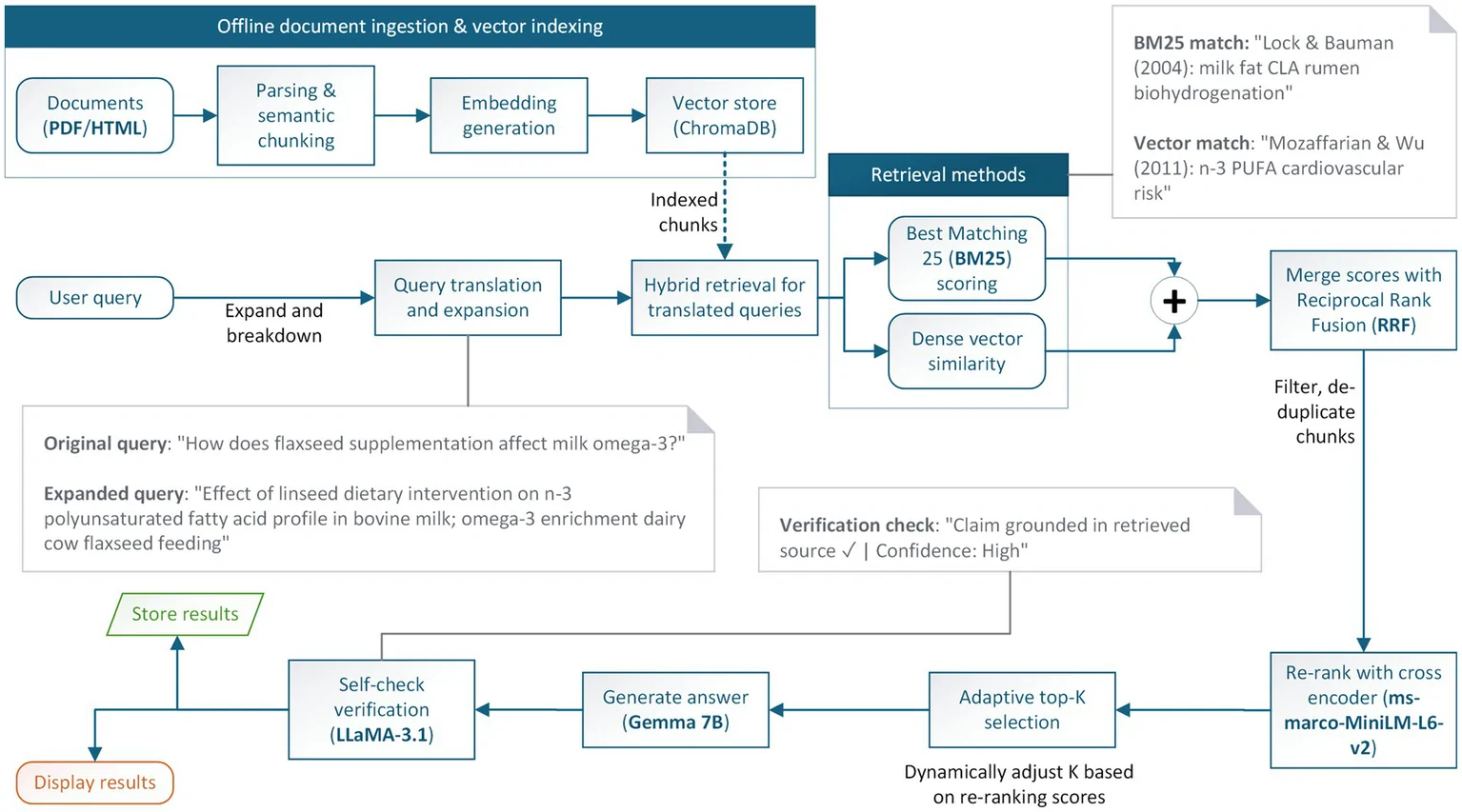
Insight nine-stage retrieval-augmented generation pipeline. The offline phase (gray background) covers document ingestion, parsing, semantic chunking, embedding generation, and vector indexing in ChromaDB. The online inference phase (white background) processes each user query through query translation and expansion (Stages 2–3, Gemma-7B), hybrid dense–sparse retrieval combining Nomic vector embeddings and BM25 lexical matching (Stage 4), reciprocal rank fusion and SHA-256 deduplication (Stage 5), cross-encoder reranking using ms-marco-MiniLM-L6-v2 (Stage 6), adaptive top-K document selection (Stage 7), evidence-grounded response generation (Stage 8, Gemma-7B), and independent self-verification (Stage 9, LLaMA-3.1-8B). Annotation boxes at Stages 2–3, 4, and 9 illustrate representative intermediate outputs at each step.

The INSIGHT system implements a nine-stage online RAG pipeline, preceded by an offline document ingestion and vector indexing phase, optimized for scientific literature retrieval and synthesis (Figure 1). The architecture comprises: (1) document ingestion, (2) query translation, (3) query expansion, (4) hybrid retrieval, (5) de-duplication, (6) cross-encoder reranking, (7) adaptive document selection, (8) LLM-based reasoning, and (9) self-verification. Document ingestion (Stage 1) is performed offline and serves as a prerequisite to online inference. Scientific documents, in Portable Document Format (PDF) or Hypertext Markup Language (HTML), are parsed and semantically segmented into retrievable units that are converted into dense vector representations and indexed for downstream retrieval. These embeddings are stored in a persistent vector database (ChromaDB) using hierarchical navigable small-world (HNSW) indexing, enabling efficient similarity-based retrieval at query time. During online inference, user queries undergo query translation and expansion (Stages 2–3) using Gemma 7B, which decomposes and semantically enriches the original query to improve retrieval coverage. Expanded queries are processed through a hybrid retrieval strategy (Stage 4) that combines dense vector similarity search (Nomic embeddings queried against ChromaDB) with sparse lexical matching using Best Matching 26 (BM25). Retrieval outputs from both methods are merged using reciprocal rank fusion, followed by de-duplication of overlapping chunks, using SHA-256 hashing (Stage 5). Retrieved candidates are subsequently reranked using a cross-encoder model: Microsoft MAchine Reading COmprehension (ms-marco) dataset, using a compact transformer language model (MiniLM) with six transformer layers (L6) version 2 (ms-marco-MiniLM-L6-v2) in Stage 6, after which an adaptive top-K selection mechanism dynamically determines the number of retained evidence chunks based on reranking confidence (Stage 7). The final answer is generated using LLM-based reasoning (Gemma 7B; Stage 8), conditioned on the selected evidence, and then undergoes an independent self-verification step using LLaMA-3.1 (Stage 9) to assess internal consistency and completeness, before results are stored and presented to the user. Together, these stages form an integrated RAG workflow that supports accurate, evidence-grounded scientific question answering while maintaining transparency. The implementation of the independent self-verification step (Stage 9) aligns with the Self-RAG framework proposed by Asai et al. (45), which demonstrates that ‘self-reflection’ tokens allow a model to autonomously critique and grade its own citations for relevance and factual support. By decoupling response generation (Gemma-7B) from verification (LLaMA-3.1), the INSIGHT system addresses ‘attributability’ challenges where models might otherwise conflate pre-trained parametric knowledge with retrieved context.

#### Document processing and parsing pipeline

Raw document processing began with the ingestion of PDF and HTML using advanced parsing algorithms designed to preserve structural information and handle the complex formatting standard in scientific publications. Section identification utilized regular expressions combined with machine learning classifiers trained to recognize common patterns in scientific literature, including abstract, methods, results, and discussion sections. Table detection employed computer vision techniques to identify tabular data, followed by structured parsing to extract numerical values, units, and associated metadata. This approach was particularly important given that quantitative nutrition data are frequently presented in tabular format.

#### Hallucination mitigation and freshness

To ensure accuracy and currency of information, INSIGHT combines multiple complementary strategies to minimize hallucinations in natural language generation (NLG). Hallucinations are unintended outputs that degrade system performance and fail to meet user expectations in real-world scenarios. These strategies include retrieval augmentation, a method that introduces an external retriever into the system to reduce conversational hallucinations (46). Additionally, it uses answer-with-citations formatting and explicit refusal or uncertainty expression when evidence is insufficient. By design, RAG architectures help address parametric knowledge bias—the tendency of language models to prioritize outdated memorized knowledge over newly retrieved information—by conditioning generation on external documents rather than relying solely on the model’s training data. This multi-layered approach helps maintain the reliability and relevance of the system’s outputs, while acknowledging the limitations of available evidence (46).

### Data, ontologies, and normalization

#### Data collection and source identification

The INSIGHT system’s knowledge base was developed through a structured acquisition pipeline designed to ensure broad, representative coverage of peer-reviewed literature across animal nutrition, feed composition, and human dietary research. Published articles were retrieved through a multi-source search strategy combining three complementary approaches: (1) automated harvesting through publisher application programming interface (API) queries targeting major scientific publishers including Elsevier, Springer, and Wiley; (2) systematic retrieval from open-access repositories including PubMed Central and AGRIS, the global information system for agricultural science and technology maintained by the Food and Agriculture Organization of the United Nations; and (3) targeted manual selection of seminal works identified through citation tracking and expert knowledge of foundational literature in ruminant nutrition, feed science, and nutritional biochemistry. This multi-tiered approach mitigated potential gaps arising from database limitations, language variations, or inconsistent indexing across repositories, thereby supporting the development of a comprehensive and representative knowledge base. All candidate documents were evaluated against explicit inclusion and exclusion criteria prior to incorporation into the corpus. Inclusion criteria required that documents be peer-reviewed publications reporting quantitative data on animal diets, feed ingredient characteristics, nutrient composition of animal-source foods, or measurable effects of production practices on food quality outcomes with relevance to human nutrition. Exclusion criteria encompassed: review articles and meta-analyses that did not present original primary data; studies lacking a clearly described experimental design or reproducible methods; publications with insufficient quantitative data to support evidence synthesis; and publications predating 2000 with limited methodological documentation, except where a pre-2000 publication represented a seminal methodological or conceptual contribution to the field. Studies lacking a clear experimental design, reproducible methods, or sufficient data for synthesis were excluded to maintain the integrity and reliability of the database. The temporal coverage of the resulting knowledge base is centered on literature published from 2000 to 2024, reflecting the period during which modern molecular and compositional analytical methods became standardized across the relevant subfields, with selective inclusion of foundational pre-2000 publications where these represent essential methodological or conceptual references. Metadata including publication year, study species, intervention type, and primary outcomes were cataloged to enable efficient retrieval and downstream incorporation into the INSIGHT analytical framework. The resulting knowledge base comprises approximately 4,000 PDF documents after duplicate removal, representing a comprehensive cross-section of research on livestock production, feed and ingredient characteristics, animal-source food quality, and their implications for human nutrition. Collectively, this curated repository provides a robust, evidence-based foundation for the INSIGHT system, enabling the generation of contextually relevant insights across the interface of animal science and human dietary outcomes. Database and API queries were executed between [Mar/2025] and [Dec/2025]. Retrieved records were screened in two steps: automated metadata filtering (title, abstract, keyword, and journal fields) followed by manual relevance screening against the inclusion and exclusion criteria.

**Figure 2:**
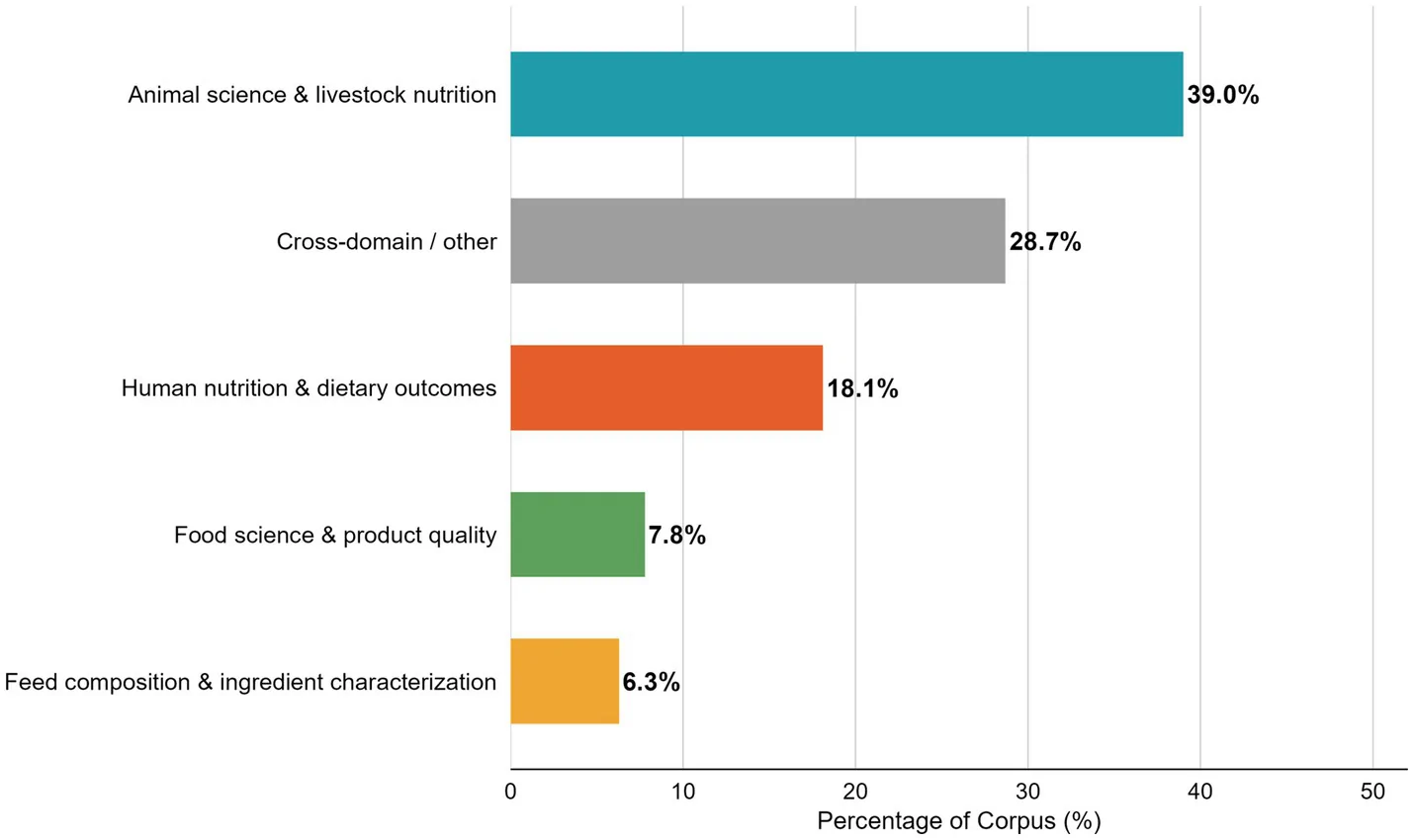
Approximate thematic distribution of the INSIGHT knowledge base.

To characterize the thematic composition of the knowledge base, all references were cataloged in EndNote (Clarivate Analytics) and their metadata, including titles, abstracts, keywords, and journal names, were analyzed using automated keyword and abstract matching. Each document was assigned to one of four primary thematic domains based on the highest-scoring match against predefined domain-specific keyword sets, with title matches weighted more heavily than abstract or keyword matches to prioritize the primary focus of each paper. The resulting approximate distribution across the classified documents is illustrated in Figure 2: animal science and livestock nutrition (~39%), encompassing studies on dietary interventions, production system effects, and physiological responses in cattle, dairy cows, sheep, poultry, swine, and aquaculture species; feed composition and ingredient characterization (~6%), including studies on the nutritional properties of forages, concentrates, supplements, and additives; human nutrition and dietary outcomes related to animal-source foods (~18%), including studies linking animal product composition to human health endpoints such as cardiovascular risk, micronutrient status, and immune function; and food science and product quality (~8%), including studies on the compositional, sensory, and functional properties of meat, milk, and eggs as influenced by production practices. An additional ~29% of documents were classified as cross-domain, spanning two or more thematic areas, a feature that reflects the inherently interdisciplinary nature of the INSIGHT knowledge base and the system’s specific purpose of bridging animal production and human nutrition evidence. This classification was performed using automated text matching and individual paper assignments carry some uncertainty; formal quantified reporting with manual expert validation is planned for subsequent versions of the system.

#### Semantic chunking and embedding generation

**Figure 3:**
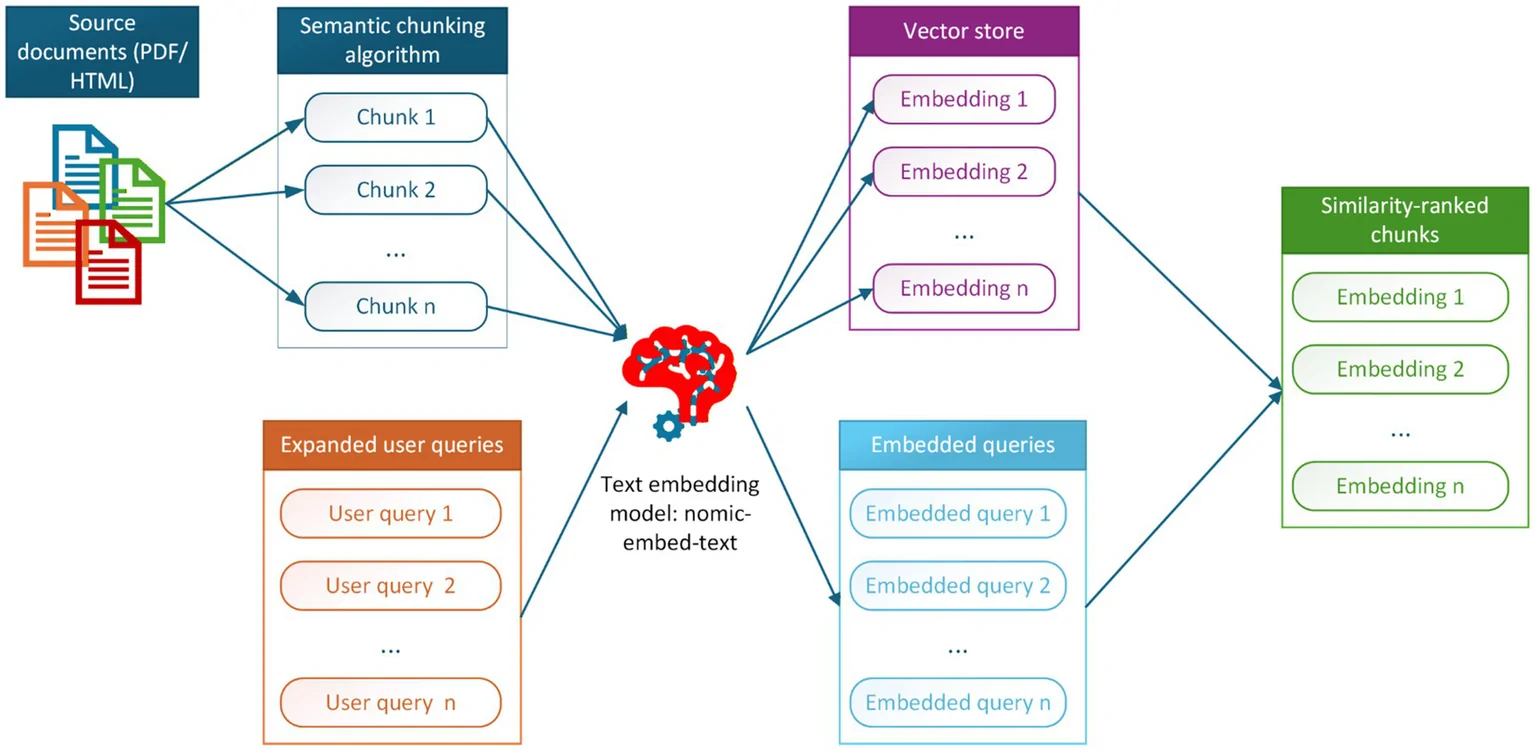
Document ingestion, semantic chunking, and embedding pipeline. PDF and HTML documents are parsed and segmented into 1,024-token chunks with 120-token overlap to preserve contextual continuity across boundaries. Each chunk is processed by the Nomic Embed Text model to generate dense vector representations that capture semantic meaning. Embeddings are stored in ChromaDB using hierarchical navigable small-world (HNSW) indexing for efficient approximate nearest-neighbor retrieval. User queries undergo the same embedding process, ensuring that both documents and queries reside in a shared vector space for direct cosine similarity comparison. The overlap design (shown in the detail inset) prevents important information from being fragmented at chunk boundaries.

To operationalize the offline ingestion and indexing stage of the RAG pipeline (Figure 3), the system implements a strategic document chunking approach using recursive character-based text splitting, dividing extracted PDF content into 1,024-token segments with 120-token overlap between consecutive chunks. This overlap design ensures contextual continuity across chunk boundaries, preventing important information from being fragmented at split points. The overlap allows the system to maintain semantic coherence, ensuring that sentences or concepts split between chunks remain interpretable when retrieved independently. Each chunk is processed by the Nomic Embed Text model, which transforms the textual content into dense vector representations that capture its semantic meaning. These embeddings enable the system to perform similarity-based retrieval by mapping documents into a high-dimensional vector space where semantically related content clusters together. The embeddings are optimized for cosine similarity calculations within ChromaDB’s HNSW index, which provides efficient approximate nearest-neighbor search with sublinear query time complexity, making the retrieval process scalable even for large document collections. User queries undergo the same embedding process, ensuring that both document chunks and expanded queries reside in a shared vector space, enabling direct similarity comparison and ranking as illustrated in Figure 3.

#### Indexing and retrieval architecture

ChromaDB serves as the persistent vector store, implementing a singleton connection pattern that ensures efficient resource management throughout the system’s lifecycle. The database architecture supports both dense vector retrieval and metadata filtering, with HNSW indexing providing sublinear query time complexity that scales effectively as the document collection grows. The ingestion pipeline optimizes throughput through batch processing with a batch size of 10 documents and employs a producer-consumer multiprocessing pattern to handle large document collections efficiently. During retrieval, the system executes a hybrid architecture that combines dense and sparse methods: dense retrieval leverages Nomic embeddings to compute cosine similarity between query and document vectors, retrieving the top-10 most similar documents for each expanded sub-query. Simultaneously, BM25 implements sparse lexical matching based on term frequency and inverse document frequency to capture exact keyword matches. The results from both retrieval methods are then merged using Reciprocal Rank Fusion with a smoothing constant of *k* = 60, which aggregates rankings without requiring score normalization and provides robust performance across different retrieval strategies. This hybrid strategy follows the optimization path established by Stuhlmann et al. (47), who demonstrated that combining BM25 lexical matching with dense vector similarity optimally balances precision and recall in large-scale biomedical corpora. Furthermore, the subsequent use of a cross-encoder for reranking (Stage 6) addresses the ‘noise’ issues inherent in initial retrieval steps, a component found to be essential for high-fidelity scientific question-answer in complex document environments.

### INSIGHT system evaluation

#### Document corpus sampling and grouping

To evaluate the performance of the INSIGHT system across diverse literature contexts, a stratified evaluation framework was developed using the full document corpus. From the complete collection of approximately 4,000 peer-reviewed papers, 282 documents were randomly selected to ensure broad representation of research topics within animal nutrition and human health. These documents were then organized in a second, separate step into semantically coherent groups based on shared keywords and topical similarity, with each group containing approximately 10 papers addressing related research questions. The two steps served distinct purposes: random selection ensured that the evaluation subset was representative of the full corpus without systematic topical bias, while the subsequent post-hoc grouping by topic ensured that evaluation questions could be meaningfully answered using the papers within each group, mimicking realistic information retrieval scenarios where relevant evidence is distributed across a focused set of related publications. This grouping approach ensured that evaluation questions could be meaningfully answered using the papers within each group, mimicking realistic information retrieval scenarios where relevant evidence is distributed across a focused set of related publications. The initial grouping procedure yielded 42 document sets (i.e., groups). To ensure robust statistical evaluation, groups were excluded if they contained fewer than 4 papers, as smaller sets provide insufficient context for meaningful retrieval assessment. Additionally, groups exhibiting non-informative retrieval patterns were excluded from analysis under two conditions: (a) groups with fewer than 4 papers, which provide insufficient context for meaningful retrieval assessment; and (b) groups exhibiting perfect precision and recall across all questions, indicating that retrieval was trivially constrained, essentially measuring keyword matching rather than semantic retrieval, because the group’s topical scope was so narrowly defined that any retrieval approach would succeed. It is acknowledged that this exclusion introduces a conservative downward bias in the reported performance metrics, since the excluded perfect-score groups would have increased overall averages if included. The reported metrics in the main text should therefore be interpreted as a conservative estimate of system performance under challenging, semantically heterogeneous retrieval conditions. After applying these quality control filters, 26 document groups remained, representing diverse topical domains within the nutrition research literature and providing a rigorous testbed for system evaluation. Formally, groups were defined by shared keyword overlap and topical similarity across titles, abstracts, and keywords; this grouping affected only which questions could be answered within a group and had no bearing on retrieval, which always operated over the full ~4,000-document corpus.

#### Ground-truth generation using deep research

For each of the 26 document groups, a controlled comparative analysis was conducted using the group’s curated set of peer-reviewed research papers (i.e., PDF documents). Perplexity’s Deep Research was used to generate 25 question–answer pairs derived exclusively from the paper within each group. For each question, Deep Research also identified the subset of papers supporting the answer, thereby defining the *ground-truth relevant documents* (i.e., number of relevant documents). Across the 26 groups, a minimum of 15 of the 25 questions were answerable using the available papers, ensuring sufficient question coverage per group. This procedure ensured that all correct answers were fully contained within the predefined corpus and could be objectively verified against source material, establishing a closed and reproducible evaluation environment across multiple independent document sets. Critically, the ground-truth document assignments generated by Deep Research were not purely AI-generated opinions; they were document identifications made within a fixed, closed corpus of verifiable peer-reviewed papers, meaning that any reader can independently confirm the correctness of individual ground-truth assignments by consulting the cited source documents directly. This closed-corpus verifiability substantially mitigates automation bias concerns inherent in open-ended AI evaluation designs. Expert human validation of a randomly sampled subset of question & answer pairs is identified as a priority for future evaluation cycles.

#### Dual querying of AI systems

For each document group, each question was independently posed to two AI systems: (i) the INSIGHT RAG system and (ii) Anthropic’s Claude Sonnet 4.6. INSIGHT was allowed to retrieve and cite documents from the full ~4,000-document knowledge base (not from the limited 282-document evaluation subset or the specific group of ~10 papers), yielding the number of retrieved documents. This is an important distinction: the retrieval task required INSIGHT to identify the correct 2–5 relevant documents from a corpus of ~4,000, representing a genuinely challenging needle-in-a-haystack retrieval problem rather than a trivially constrained selection from a pre-filtered set. Precision and recall were then calculated by comparing the documents retrieved from the full corpus against the ground-truth relevant documents identified within each group. In contrast, Claude Sonnet 4.6 operated without access to the reference papers and relied solely on its internal parametric knowledge. This dual-query design enabled a direct comparison between a domain-grounded RAG-based system and a general-purpose LLM operating in a closed-book setting, replicated across 26 independent literature contexts. For INSIGHT, the *number of relevant retrieved documents* was determined by identifying the overlap between the retrieved documents and the ground-truth relevant documents. The graphical relationships among the number of relevant documents, retrieved documents, and relevant retrieved documents, from which precision, recall, and F1-score are derived, are illustrated in Supplementary Figure S1. This retrieval-versus-parametric asymmetry is the intended object of study rather than a confound: Claude Sonnet is used as a closed-book reference point to isolate the contribution of retrieval grounding, not as an equivalent retrieval system.

#### Calculation of evaluation metrics

Precision, recall, and the F1-score are widely used performance metrics for evaluating the adequacy of AI models, particularly in classification, information retrieval, and decision-support systems. Precision quantifies the reliability of positive predictions by measuring the proportion of retrieved or predicted instances that are truly relevant. In contrast, recall measures completeness by assessing the model’s ability to identify all relevant instances in the dataset. These two metrics often reflect a fundamental trade-off: models optimized for high precision may miss relevant cases (low recall), whereas models optimized for high recall may include a higher proportion of false positives (low precision). This trade-off is especially important in high-stakes applications such as medical diagnosis, risk prediction, or scientific information retrieval, where the costs of false positives and false negatives differ substantially. The F1-score provides a single summary statistic that balances precision and recall by computing their harmonic mean. Unlike the arithmetic mean, the harmonic mean penalizes extreme imbalance between precision and recall, ensuring that a high F1-score is achieved only when both metrics are reasonably high. Consequently, the F1-score is commonly used when class distributions are imbalanced or when both false positives and false negatives are consequential. In applied AI systems, reporting precision, recall, and F1-score together offers a more transparent and informative assessment of model adequacy than overall accuracy alone, particularly in contexts where accuracy can be misleading due to class imbalance or asymmetric error costs. For this evaluation, precision (Equation 1), recall (Equation 2), and F1-score (Equation 3) were calculated for each question within each document group, yielding distributions of performance metrics across diverse retrieval contexts.


$$\text{Precision}=\frac{\mathrm{TP}}{\mathrm{TP}+\mathrm{FP}}=\frac{\text{number of relevant retrieved documents}}{\text{number of retrieved documents}}\;$$ (1)

$$\text{Recall}=\frac{\mathrm{TP}}{\mathrm{TP}+\mathrm{FN}}=\frac{\text{number of relevant retrieved documents}}{\text{number of relevant documents}}$$ (2)

$$F1\;\text{Score}=\frac{2\times \text{Precision}\times \text{Recall}}{\text{Precision}+\text{Recall}}$$ (3)

Where TP is true positive, FP is false positive, and FN is false negatives.

### Statistical analyses

Performance metrics (precision, recall, and F1-score) were calculated for each question within each of the 26 document groups. Because metrics were computed independently across questions and groups, a hierarchical analysis strategy was employed to account for both question-level (within-group) and group-level (between-group) variability (48).

#### Within-group and between-group analysis

To assess whether retrieval performance varied systematically across document groups, a Generalized Linear Mixed Model (GLMM) was implemented using PROC GLIMMIX in SAS (SAS Institute Inc., Cary, NC). Given that precision, recall, and F1-score are proportions bounded by [0, 1], a Beta distribution with a logit link function was specified. To accommodate the Beta distribution’s requirement for values strictly within the open interval (0, 1), a Smithson and Verkuilen (49) transformation was applied, as shown in Equation 4:


$$y^{\prime }=\frac{y\times (N-1)+0.5}{N}$$ (4)

Where y’ represents the transformed proportion (precision, recall, and F1 score), y represents the original proportion, and N is the sample size (i.e., 614 questions). Document group was modeled as a fixed effect to test whether performance differed across the 26 independent literature contexts. This analysis provides insight into the robustness of INSIGHT’s retrieval strategy across diverse topical domains within the nutrition research literature. The transformed proportions (precision, recall, and F1-score) were used only for this analysis.

#### Descriptive statistics and macro-averaging

Treating each question as an independent evaluation unit, descriptive statistics for precision, recall, and F1-score—including the mean, standard deviation, minimum, maximum, median, and 95% confidence intervals for the mean—were computed across all questions using PROC MEANS in SAS (SAS Institute Inc., Cary, NC) and the nontransformed proportions. This macro-averaged approach provides an assessment of both central tendency and variability of model performance across heterogeneous queries and literature contexts, rather than allowing results to be dominated by a small subset of questions or groups (48).

#### Baseline performance testing

To formally evaluate whether model performance exceeded a non-informative baseline, question-level metrics were first aggregated within each group to obtain group-level mean precision, recall, and F1-score. These group-level averages (*n* = 26) were then subjected to one-sample t-tests using PROC TTEST in SAS (SAS Institute Inc., Cary, NC) and the nontransformed proportions. The null hypothesis tested whether the grand mean (across groups) for each metric equaled 0.5, which represents a practical reference point corresponding to performance no better than a naïve or minimally informative classifier in balanced binary decision settings. A one-sided alternative hypothesis (mean > 0.5) was specified to test whether the model demonstrated predictive capability beyond this baseline. Because precision (Equation 1), recall (Equation 2), and F1-score (Equation 3) are bounded on the interval [0, 1], 0.5 serves as a conservative and interpretable internal significance anchor under a balanced relevance assumption, rather than as a claim about an externally standardized retrieval benchmark.

#### Contextual comparison to published benchmarks

For contextualization purposes only, the observed group-averaged performance metrics were compared descriptively to those reported by Stuhlmann et al. (47), who evaluated a biomedical RAG system using the BioASQ question-answering benchmark built on PubMed. They conducted a two-phase evaluation: first, a component analysis on a 10% randomly sampled subset (2.4 M documents) comparing retrieval methods (BM25, BioBERT, MedCPT, and a hybrid BM25-MedCPT approach) and storage systems (Elasticsearch, FAISS, MongoDB); second, a final system evaluation on the whole 24 M PubMed corpus examining the effect of retrieval depth (20, 50, 100 documents). Their evaluation used a single, unified corpus with questions drawn from BioASQ’s expert-annotated question sets, where ground-truth relevant documents were predefined by domain experts. The hybrid retriever (BM25 + MedCPT cross-encoder reranking) with 50 retrieved documents achieved their best end-to-end performance: accuracy = 0.90, recall = 0.90, precision = 0.89, F1 = 0.90. Their analysis focused on optimizing retrieval depth and balancing response time against answer quality within a single, static evaluation framework. For a hybrid BM25-MedCPT retrieval system evaluated on the BioASQ benchmark, they reported precision = 0.89, recall = 0.86, and F1-score = 0.86. Therefore, a direct statistical comparison was not performed due to fundamental differences in evaluation design, including corpus composition, ground-truth generation methodology, and sampling strategy. Instead, this comparison serves solely to situate INSIGHT’s performance within the broader landscape of biomedical RAG systems. Although the INSIGHT system’s recall (0.62) is lower than the optimized hybrid peaks reported by Stuhlmann et al. (47), it remains competitive with the performance of advanced agentic frameworks like those evaluated by Ase et al. (30), who noted that increasing system complexity through multi-agent verification can introduce trade-offs in absolute recall while significantly enhancing the reliability and safety of the output. This trade-off is particularly relevant in nutrition and medical domains where factual precision and verifiability are prioritized over exhaustive retrieval.

## Results and discussion

### Within-group and between-group analysis

The analysis of the INSIGHT RAG system’s performance across 26 experimental groups revealed considerable heterogeneity in results. A GLMM using a Beta distribution and logit link function was employed to account for the bounded nature of the performance metrics. The Type III Tests of fixed effects indicated a highly significant effect of the group factor on precision, recall, and F1-scores (*p* < 0.0001). The high denominator degrees of freedom (588) confirm that the model effectively utilized the individual question-level variance within each group to derive its estimates. This indicates that the performance differences observed between groups are not merely artifacts of random variation among questions, but rather reflect distinct performance levels inherent to the different system configurations or task types assigned to each group. The significant variability observed across document groups (*p* < 0.0001) underscores the ‘adaptability’ requirement highlighted by Asai et al. (45), noting that RAG performance is susceptible to the semantic complexity of the query domain. Our findings suggest that while a hybrid pipeline is robust, particular topical clusters in nutrition research represent ‘hard’ retrieval tasks that benefit from the iterative, agentic approach proposed by Ase et al. (30) to improve diagnostic-level accuracy.

The significant *p*-value from the PROC GLIMMIX analysis demonstrates that the INSIGHT RAG system did not perform uniformly across all tested scenarios. This variability suggests that the interaction between the retriever and the LLM is highly sensitive to the specific context or domain of the queries within each group. The fact that groups are statistically distinct implies that while the INSIGHT RAG system may excel in certain query sets, it might encounter significant bottlenecks in others. By analyzing the data at the question level within a mixed model, we have accounted for the “noise” of unvetted questions. The significant results prove that the “signal”—the impact of the group configuration—is strong enough to overcome the variance of individual questions. These results provide a robust internal baseline. Having established that the groups differ significantly, we can now identify which configurations represent the system’s “peak” performance and which require further optimization to meet established benchmarks for nutritional conditions.

The significant between-group performance variation (*p* < 0.0001) is one of the most practically informative findings of this study, as it reveals specific structural characteristics of the retrieval task that drive performance heterogeneity across literature domains. We propose four mechanistic categories of explanation. First, vocabulary mismatch and cross-domain terminology divergence represent a fundamental challenge for hybrid retrieval in an interdisciplinary corpus. Groups spanning both animal science and human nutrition literature frequently employ divergent nomenclature for functionally analogous concepts, e.g., ‘omega-3 supplementation in ruminants’ versus ‘n-3 polyunsaturated fatty acid (PUFA) and cardiovascular risk’, creating semantic distance in the embedding space that neither dense vector retrieval nor BM25 lexical matching fully resolves. When the same biological phenomenon is described using discipline-specific terminology in different parts of the corpus, retrieval systems that lack explicit cross-domain concept mapping will systematically underperform on queries that require integrating evidence from both subfields. Second, conceptual density and semantic structure likely contribute to performance differences across topically distinct groups. Groups addressing mechanistically complex topics, e.g., rumen biohydrogenation pathways, methyl donor metabolism, or antioxidant enzyme cascades, present more challenging retrieval tasks because relevant evidence is distributed across tightly interrelated passages rather than concentrated in topically coherent sections. In such groups, a single query may require evidence from multiple mechanistic steps, each described in separate documents using specialized terminology, which reduces both precision and recall relative to groups where the relevant evidence is more localized. Third, limitations of the Nomic Embed Text embedding model may differentially affect performance across groups. While Nomic Embed Text performs strongly on general scientific text, it may underperform on highly specialized agricultural and ruminant nutrition terminology that was underrepresented in its pretraining corpus. Groups whose terminology departs most substantially from the general scientific register represented in large pretraining datasets are therefore likely to show lower retrieval performance, particularly for queries involving species-specific production terminology or feed ingredient nomenclature. Fourth, terminology normalization gaps amplify all of the above effects. In the absence of controlled vocabulary alignment with AGROVOC or standardized feed ingredient ontologies, synonymous terms used across subfields are not mapped to shared concepts, and variant spellings, abbreviations, and disciplinary conventions are treated as distinct tokens. This reduces recall for cross-disciplinary queries and contributes to the observed between-group variance. Consistent with this interpretation, the document groups with the highest F1-scores in the present evaluation tended to involve topics with well-standardized and relatively constrained terminology, such as specific micronutrient supplementation trials (e.g., selenium, vitamin E, or chromium supplementation in dairy cattle), where the relevant literature employs consistent nomenclature across animal science and human nutrition subfields. Groups with the lowest F1-scores, by contrast, tended to involve broader or more mechanistically complex topics where terminology varied substantially across papers. These patterns support the terminology-normalization hypothesis as a primary driver of performance heterogeneity and underscore the value of the AGROVOC integration and knowledge graph approaches described in the *Opportunities* section as targeted interventions to address this limitation. These four explanations are offered as candidate hypotheses rather than experimentally confirmed causes; each is a proposed mechanism that warrants targeted future testing, as outlined in the Opportunities section.

### Descriptive statistics of macro-averaging and baseline performance testing

Table 1 presents the average and standard deviation for each group, as well as the overall averages and standard deviations for precision, recall, and F1-score. The analysis of the 26 group averages yielded a mean precision of 0.7675 and a standard deviation of 0.1183, suggesting that the INSIGHT RAG system’s performance was significantly higher than the 0.5 baseline (*p* < 0.0001). For recall, the mean was 0.6191 with a standard deviation of 0.1469, and this result was significantly greater than the baseline (*p* = 0.0002). The mean of F1-score was 0.6737 with a standard deviation of 0.1005, demonstrating highly significant superior performance compared to the 0.5 threshold (*p* < 0.0001).

**Table 1:** Nontransformed average evaluation metrics for within and between document groups^1^.

Group	*n*	Precision		Recall		F1-Score	
		Average	SD	Average	SD	Average	SD
0	25	0.927	0.205	0.408	0.151	0.550	0.155
1	25	0.893	0.263	0.813	0.298	0.821	0.263
2	25	0.913	0.205	0.693	0.266	0.751	0.198
3	26	0.518	0.292	1.000	0.000	0.639	0.237
4	25	0.652	0.281	0.673	0.226	0.622	0.192
5	25	0.727	0.385	0.397	0.269	0.486	0.277
6	24	0.597	0.274	0.508	0.229	0.521	0.205
7	25	0.657	0.362	0.419	0.264	0.479	0.264
10	25	0.863	0.294	0.430	0.225	0.547	0.213
11	26	0.856	0.230	0.737	0.227	0.755	0.175
12	23	0.899	0.206	0.783	0.253	0.801	0.192
13	25	0.700	0.373	0.680	0.360	0.675	0.343
14	21	0.691	0.303	0.500	0.307	0.551	0.268
15	25	0.775	0.312	0.587	0.241	0.632	0.218
16	24	0.788	0.285	0.605	0.247	0.658	0.229
17	25	0.677	0.337	0.493	0.270	0.548	0.270
18	25	0.776	0.254	0.528	0.210	0.584	0.154
20	25	0.809	0.292	0.553	0.267	0.609	0.213
21	24	0.803	0.233	0.663	0.262	0.678	0.174
23	26	0.654	0.313	0.705	0.310	0.644	0.271
26	25	0.870	0.201	0.580	0.277	0.652	0.211
29	25	0.692	0.292	0.780	0.295	0.673	0.207
32	15	0.767	0.234	0.522	0.188	0.604	0.171
33	25	0.887	0.258	0.660	0.332	0.717	0.274
35	15	1.000	0.000	0.789	0.240	0.862	0.158
37	15	0.643	0.315	0.589	0.308	0.564	0.232
Average		0.770		0.619		0.639	
SD		0.118		0.147		0.100	

^1^*n* is the number of questions, and SD, standard deviation.

It is important to note that a strong document-level grounding does not necessarily translate into superior perceived answer quality. When only a subset of relevant documents is retrieved, the generation component operates with incomplete contextual information, which can reduce the completeness of answers or the depth of synthesis, while maintaining factual correctness. Prior studies have shown that retrieval-augmented systems may therefore underperform in answer-quality evaluations when retrieved context is incomplete, fragmented, or noisy, even though the retrieved evidence itself is accurate (31, 32).

### Contextual comparison to published benchmarks

When group-averaged metrics were compared to Stuhlmann’s et al. (47) reported benchmarks using one-sample t-tests, the INSIGHT RAG system exhibited significantly lower precision (mean = 0.77 vs. 0.89; *t*₂₅ = −5.15, *p* < 0.0001), recall (mean = 0.62 vs. 0.86; *t*₂₅ = −8.36, *p* < 0.0001), and F1-score (mean = 0.64 vs. 0.86; *t*₂₅ = −11.20, *p* < 0.0001). However, these statistical differences should not be interpreted as evidence of inferior system performance, as the comparison violates fundamental assumptions of statistical inference due to critical methodological differences between the two studies. First, corpus structure differs markedly: Stuhlmann et al. (47) evaluated retrieval performance on a single, unified PubMed corpus, whereas the INSIGHT RAG system was assessed across 26 semantically distinct document groups to evaluate robustness across diverse literature contexts. Second, ground-truth generation differed: Stuhlmann et al. (47) used BioASQ’s expert-curated question sets with predefined relevant documents, whereas the INSIGHT RAG system used Perplexity Deep Research to generate questions and identify relevant papers within each group, leading to different definitions of document relevance. Third, sampling and variance structures are incomparable: Stuhlmann’s et al. (47) single-corpus design produces statistical properties different from INSIGHT’s replicated multi-group evaluation, making a formal statistical comparison invalid without access to their question-level raw data and within-corpus variance. Fourth, the evaluation objectives differ: Stuhlmann et al. (47) optimized retrieval depth and response time within a fixed corpus, whereas the INSIGHT RAG system prioritized assessing retrieval consistency across heterogeneous topical domains. The observed numerical differences likely reflect these design choices—particularly INSIGHT’s more conservative retrieval strategy across smaller, semantically constrained document groups and the use of automated ground-truth generation—rather than fundamental differences in system capability. Consequently, although both studies demonstrate the effectiveness of hybrid retrieval strategies for biomedical question-answering, the statistical comparison is methodologically invalid. The results from Stuhlmann et al. (47) are presented solely to contextualize INSIGHT’s performance within contemporary biomedical RAG research, acknowledging that hybrid retrieval architectures consistently demonstrate strong performance across different evaluation frameworks, even when absolute metric values differ due to evaluation design. These external figures are therefore offered only as approximate context from the broader literature; a direct quantitative comparison is not methodologically valid given the differences in corpus composition, ground-truth construction, and evaluation protocol.

The results confirm that the INSIGHT RAG pipeline was objectively effective, consistently outperforming the 0.5 baseline across all metrics. Using the group averages provides a robust “macro-average” that accounts for the inherent difficulty variance of the biomedical queries. Comparing these results with those of Stuhlmann et al. (47) reveals that the INSIGHT RAG system aligns well with standard retrieval methods but has room to reach their optimized “Hybrid” peaks. The precision of our system (0.7675) is quite robust. While our system’s precision (0.77) and recall (0.62) are lower than the optimized hybrid peaks of Stuhlmann et al.'s (47) (0.89 and 0.86, respectively), our results are highly competitive with their reported BM25-only (precision of 0.83) and BioBERT (recall of 0.63) baselines. The performance gap likely reflects their use of an NVIDIA A30 GPU to process a retrieval depth of 50 documents before reranking, a configuration that maximizes recall but increases computational latency—a trade-off the INSIGHT system balances through its on-premises, local deployment architecture.

The use of 26 groups with roughly 25 questions each mirrors the systematic approach in Stuhlmann et al. (47), which emphasized evaluating retrieval depth and response-time trade-offs. Stuhlmann et al. (47) noted that increasing retrieval depth beyond 50 documents often leads to diminishing returns in accuracy. Our lower standard deviations (particularly 0.1005 for F1-Score) suggest that the INSIGHT RAG system provides a consistent baseline of performance across different biomedical tasks.

#### Contextualizing LLM performance across nutrition AI applications

The INSIGHT RAG system’s performance (precision = 0.77, recall = 0.62, F1 = 0.67) demonstrates effective retrieval across a complex multi-group evaluation framework. While direct statistical comparison across different task types is methodologically invalid, examining LLM performance in related nutrition domains provides valuable interpretive context for understanding the scope and limitations of AI-driven nutritional assessment systems. Ase et al. (30) evaluated three frontier LLM (Claude Opus 4.5, Gemini 3 pro, GPT-5.1) on binary dietary classification of 1,992 Polish food items against NOVA and WHO nutritional frameworks, achieving 90.3–94.2% agreement with expert-validated consensus, a substantially higher concordance than INSIGHT’s retrieval metrics. However, this performance gap reflects fundamental differences in task complexity rather than system capability. Binary classification of predefined food items against established criteria represents a bounded decision space with clear ground truth, whereas INSIGHT’s multi-document retrieval task requires semantic matching across ~4,000 heterogeneous scientific papers, where relevance is contextual, evidence is distributed, and “correct” retrieval depends on query interpretation. Critically, Ase et al. (30) demonstrated that even in the simpler classification domain, all pairwise LLM comparisons showed statistically significant differences (*p* < 0.001), indicating that different models interpret identical nutritional criteria differently—a pattern directly analogous to our multi-group findings showing significant between-group performance variation (*p* < 0.0001). Moreover, their finding that prompt structure critically influenced classification outcomes—with structured NOVA+WHO prompts yielding very high recall (0.96–0.98) but reduced specificity (0.80–0.84), while simplified prompts achieved better balance (recall: 0.91–0.96; specificity: 0.90–0.95)—parallels INSIGHT’s observed trade-offs between retrieval depth and precision. In both systems, configuration choices determine whether the model prioritizes sensitivity (capturing all relevant items) or specificity (avoiding false positives), with no single optimal balance applicable across all use cases. Both studies independently converge on a fundamental conclusion: LLM can achieve near-expert performance on specific nutrition tasks when properly configured, but systematic biases persist across models and prompting strategies, requiring sustained human oversight for high-stakes applications. The key distinction is that INSIGHT’s RAG architecture addresses LLM limitations through transparent evidence grounding—every answer is traceable to specific retrieved documents—whereas pure parametric models lack this verifiability, even when achieving high classification agreement.

### Practical applications

Beyond its demonstrated retrieval capabilities, INSIGHT is designed to address concrete evidence synthesis challenges across several interconnected deployment contexts. Four practical applications are particularly relevant given the system’s cross-domain architecture and evidence-grounded design.

First, INSIGHT can serve as a first-pass evidence aggregation tool to accelerate systematic and scoping reviews in animal science and human nutrition. Empirical analyses demonstrate that a single medical systematic review requires an average of 67.3 weeks and a team of five researchers to complete (26), with comparable demands expected in animal science and nutrition, given similar literature search and synthesis burdens. By autonomously retrieving and ranking relevant documents across a curated corpus of approximately 4,000 peer-reviewed publications, INSIGHT can substantially reduce the manual screening burden, allowing domain experts to redirect their effort toward the higher-order tasks of evidence appraisal, quality assessment, and interpretive synthesis that require human judgment. This positions INSIGHT not as a replacement for systematic review methodology but as a precision tool that compresses the early evidence aggregation phase while maintaining transparency through explicit citation provenance.

Second, INSIGHT is uniquely positioned to bridge the feed science–human nutrition gap that currently limits translation of agricultural research into dietary guidance. The system’s cross-domain corpus and hybrid retrieval architecture enable it to respond to queries that simultaneously require knowledge of animal feeding strategies and their downstream effects on the nutritional composition of animal-source foods and human health outcomes, a class of questions that no single-domain database or general-purpose LLM can adequately address. For example, a researcher or policymaker asking how pasture-based finishing systems affect the omega-3 fatty acid profile of beef and its relevance to cardiovascular disease risk reduction must navigate literature distributed across ruminant nutrition, food science, and clinical nutrition journals. INSIGHT’s integrated corpus and ontology-bridging retrieval architecture are specifically designed to support this type of cross-domain inquiry.

Third, INSIGHT can facilitate interdisciplinary food-system research by providing a shared, evidence-grounded knowledge interface for researchers working across disciplinary boundaries. Collaborations between animal scientists, nutritionists, food scientists, and public health researchers are increasingly necessary to address complex food system challenges, including sustainable intensification, the nutritional transition in low- and middle-income countries, and the optimization of animal-source food quality for human health, but are hampered by disciplinary silos in both literature access and terminology. By integrating evidence across these domains with explicit provenance, INSIGHT can serve as a common evidentiary foundation that supports cross-disciplinary dialogue without requiring individual researchers to develop expertise in adjacent literatures.

Fourth, INSIGHT can contribute to the evidence base for nutrition policy and dietary guidance development by providing traceable, quantitative links between animal production practices and the nutrient profiles of animal-source foods. Recent shifts in dietary guidance frameworks, including the increased emphasis on animal-source foods as nutrient-dense components of the diet and the growing recognition of production-system effects on food composition, highlight the need for transparent, evidence-traceable systems that link dietary recommendations to the underlying scientific literature on which they depend. INSIGHT’s self-verification architecture and citation-linked response generation are specifically designed to support this traceability requirement, enabling policy analysts and guideline developers to interrogate the evidentiary basis of compositional claims rather than relying solely on narrative summaries.

### Opportunities for improving retrieval and generalizability

While the current INSIGHT system demonstrates effective performance in analyzing nutrition research claims within a controlled corpus, future iterations could benefit from tighter alignment with standardized agricultural vocabularies and enhanced food composition databases to improve robustness and global applicability. Resources such as FoodData Central’s Foundation Foods dataset provide per-sample variability and rich agricultural metadata, offering the granularity needed to connect production variables with nutrient distributions. However, aligning research claims to database entities remains challenging in the absence of shared vocabularies and explicit reasoning mechanisms. In addition, planned methodological work includes a component-wise ablation to quantify each pipeline stage’s contribution, a sensitivity analysis over chunk size and overlap, and controlled experiments (for example, controlled-vocabulary alignment and alternative embedding models) to test the candidate explanations for the observed cross-group performance variation.

Future versions of INSIGHT could leverage AGROVOC, a multilingual controlled vocabulary maintained by the Food and Agriculture Organization of the United Nations, as a unifying concept hub for agricultural terminology spanning feeds, species, and management practices (50). Integration of AGROVOC could enable normalization of synonymous terms across heterogeneous research corpora and databases, potentially improving both recall and precision in information retrieval and entity linking. Such capabilities would be particularly valuable for enhancing global compatibility, as AGROVOC’s multilingual framework could facilitate the processing of agricultural and nutrition research from non-English sources. Collectively, these enhancements could improve INSIGHT’s resilience to variation in terminology across agricultural systems and research traditions, supporting more comprehensive and internationally applicable nutrition research synthesis. Beyond terminology normalization, two emerging architectural directions offer substantial potential for advancing INSIGHT’s synthesis capabilities in future development cycles. The first is the integration of structured knowledge graphs with the existing RAG pipeline. Knowledge graphs encode domain entities, such as feed ingredients, metabolic pathways, nutrient outcomes, and animal species, as typed nodes connected by semantically defined relations, enabling multi-hop reasoning across linked evidence rather than relying solely on embedding similarity. Edge et al. (51) demonstrated that building an entity knowledge graph from source documents and generating community summaries across related entity clusters leads to substantial improvements over conventional vector RAG for global sensemaking queries, achieving comprehensiveness win rates of 72–83% and diversity win rates of 75–82% across two large corpora. This capacity for corpus-wide thematic synthesis is directly relevant to INSIGHT’s cross-domain challenge of integrating evidence distributed across heterogeneous literature in animal science and human nutrition. Incorporating an agricultural-nutrition knowledge graph aligned with AGROVOC and USDA FoodData Central ontologies could substantially improve INSIGHT’s ability to answer mechanistic questions spanning multiple literature subdomains. The second direction is the evolution toward fully agentic architectures in which specialized sub-agents handle distinct functions in a coordinated pipeline. Wu et al. (52) introduced AutoGen, an open-source multi-agent conversation framework in which customizable agents, each assigned a distinct role such as code writing, code execution, output verification, or human feedback solicitation, communicate through structured dialogue to accomplish complex tasks. Empirical evaluations demonstrated that multi-agent designs outperformed single-agent approaches across diverse knowledge-intensive tasks, including mathematical reasoning, retrieval-augmented question answering, and code generation, with the multi-agent coding application reducing core workflow code by more than 75% while improving task success rates. The INSIGHT pipeline already incorporates agentic elements including adaptive document selection (Stage 7) and self-verification (Stage 9), and future iterations could extend this toward a full multi-agent architecture in which retrieval, synthesis, fact-checking, and uncertainty quantification are handled by independently optimized agents coordinated through a conversation-driven orchestration layer, a natural evolutionary path for a system whose ultimate goal is transparent, verifiable, cross-disciplinary evidence synthesis. A third and perhaps most critical future development direction is the implementation of a systematic review benchmarking evaluation framework. Whereas the present study evaluated INSIGHT using document-level retrieval metrics, a rigorous end-to-end synthesis quality assessment requires comparing INSIGHT-generated syntheses with authoritative human-produced summaries. The approach recommended for this evaluation is as follows: for each candidate systematic review published in animal science or human nutrition, INSIGHT would generate a synthesis of the relevant question using only literature available in the corpus prior to the review’s publication date, thereby constructing a temporally valid comparison. The similarity between the INSIGHT-generated synthesis and the published systematic review text would then be quantified using semantic textual similarity (STS) scores alongside RAG assessment (RAGA) metrics, including faithfulness, answer relevance, context precision, and context recall, providing a principled, reproducible measure of how closely the AI-generated synthesis approximates expert human evidence integration. This systematic review benchmarking approach is identified as the primary evaluation direction for the next phase of INSIGHT development.

The INSIGHT RAG system is a functional and effective biomedical question-answer tool. The *p* < 0.001 difference when compared to the 0.86 F1-score in Stuhlmann et al. (47) suggests that your system is currently operating at a lexical or single-stage retrieval level. To reach the “Stuhlmann standard,” the next development phase should focus on the hybrid reranking strategies they found to be most effective.

### Limitations and risks

#### Study heterogeneity and meta-inference challenges

The agricultural and nutrition literature exhibits substantial heterogeneity across multiple dimensions, complicating systematic evidence synthesis and meta-inference. Experimental studies vary widely in target species (cattle, sheep, swine, poultry), breed or genetic lines, dietary compositions, management systems, tissue sampling protocols, and analytical methodologies (4). This variability extends to food composition research, where factors such as muscle cut selection, post-harvest processing conditions, cooking methods, and storage duration significantly influence measured nutritional parameters. Such heterogeneity poses fundamental challenges for automated synthesis systems that aim to generate generalizable conclusions across diverse experimental contexts. INSIGHT addresses this limitation by explicitly quantifying uncertainty and reporting ranges rather than deriving overly precise point estimates from heterogeneous data sources. When synthesizing evidence across studies with varying methodologies, the system prioritizes reporting confidence intervals, study-specific contexts, and methodological differences that may influence the interpretation of results (4). This approach acknowledges the inherent variability in biological systems while providing users with appropriate context for evaluating the applicability of synthesized evidence to their specific research or practical applications.

#### Retrieval system limitations and coverage gaps

The completeness and quality of the underlying document corpus fundamentally constrain the effectiveness of RAG systems. Despite careful curation, relevant studies—particularly recent publications or work using non-standard terminology—may be absent from the indexed literature, resulting in systematic coverage gaps. Retrieval algorithms may also fail to identify relevant passages when findings are reported in unconventional formats or described using domain-specific language that does not align with trained retrieval patterns. Importantly, INSIGHT does not have access to the open web and operates strictly within its curated document corpus. As a result, its performance reflects the coverage and representativeness of the indexed literature rather than the breadth of parametric knowledge available to general-purpose LLM. Consequently, differences observed between INSIGHT and closed-book models should not be interpreted as deficiencies in retrieval accuracy but rather as structural differences in system architecture and information access. Additionally, automated systems cannot independently evaluate the scientific validity of authors’ interpretations, assess methodological rigor, or detect fabricated or unreliable findings. While INSIGHT prioritizes peer-reviewed sources and employs hybrid retrieval strategies that combine dense semantic search, keyword-based methods, and ontology-driven query expansion, it cannot replace expert judgment or a comprehensive manual literature review when exhaustive evidence appraisal is required. In addition, document parsing fidelity was not independently quantified in the present study; a targeted parsing-error audit is planned as part of future corpus-quality validation.

#### Hallucination risks and over-generalization

LLM are known to generate plausible but factually incorrect information, particularly when synthesizing across multiple sources or extrapolating beyond available evidence (33, 46). INSIGHT mitigates these risks through explicit citation linking, conservative response generation policies, uncertainty-aware language, and human review of high-risk outputs. Despite these safeguards, users must critically evaluate all generated content and consult original sources when making consequential research or policy decisions.

#### Ethics and responsible use

The deployment of AI-enabled systems for scientific literature synthesis raises important ethical considerations that warrant explicit acknowledgment. Four dimensions are particularly relevant to the responsible use of INSIGHT in research and policy contexts. First, automation bias represents a significant risk in AI-assisted evidence synthesis. Users may over-rely on AI-generated syntheses without critically appraising the underlying retrieved evidence, particularly when outputs are presented with apparent confidence and explicit citation links. The perceived authority of a system that cites peer-reviewed sources may paradoxically reduce the critical scrutiny that those sources deserve. INSIGHT mitigates this risk through its self-verification architecture (Stage 9) and uncertainty-aware language in generated responses, but users must remain vigilant that retrieved documents may themselves contain methodological limitations, conflicting findings, or context-specific conclusions that require expert interpretation. The system is designed to support expert judgment, not to replace it. Second, human oversight is essential for high-stakes applications of INSIGHT, particularly in nutrition policy formulation, clinical dietary guidance, and public health recommendations. In these contexts, the consequences of acting on incomplete, misrepresented, or out-of-date evidence are substantial. INSIGHT should be treated as a decision-support tool that accelerates evidence aggregation and improves retrieval transparency, while final interpretive and evaluative judgments remain the responsibility of qualified domain experts. Institutional deployment of INSIGHT in policy-relevant contexts should therefore incorporate structured human-review workflows rather than relying solely on automated outputs as standalone evidence. Third, transparency by design is a foundational principle of the INSIGHT architecture. Explicit citation linking, provenance display, and the presentation of retrieved source passages alongside generated responses are built into the system specifically to enable users to interrogate, verify, and if necessary, challenge the evidentiary basis of any claim the system produces. This transparency architecture distinguishes INSIGHT from general-purpose LLM that generate responses without traceable grounding and is intended to support rather than supplant domain experts’ critical appraisal skills. Users are encouraged to follow citation links to the original sources and to independently evaluate the methodological quality of retrieved studies, regardless of the system’s ranking and selection decisions. Fourth, ongoing monitoring of system performance is necessary as the underlying corpus evolves and new evidence is added. The retrieval performance characteristics documented in the present evaluation reflect the corpus as constituted at the time of assessment; as new publications are incorporated, as disciplinary terminology evolves, and as dietary guidance frameworks shift, the system’s performance profile may change in ways that require periodic re-evaluation. Institutions deploying INSIGHT should establish protocols for regular performance auditing, corpus updating, and user feedback collection to ensure that the system’s evidence base remains current, representative, and aligned with evolving scientific consensus. Anthropic’s broader guidance on responsible AI deployment in research contexts provides a useful framework for such institutional governance considerations (33).

#### Evaluation framework

The present evaluation was intentionally designed as a controlled, proof-of-concept assessment rather than an exhaustive benchmark of large language model performance. Several limitations should therefore be considered when interpreting the results. First, the evaluation was based on a relatively small corpus of 10 peer-reviewed research papers and 25 derived questions. While this design enabled precise ground-truth definition and objective verification of document relevance, it necessarily limits the diversity of research contexts, terminology, and reasoning complexity represented in the test set. As a result, performance estimates may not generalize to broader or more heterogeneous literature collections. Second, answer-quality comparisons relied on automated adjudication using a large language model rather than human domain experts. Although prior studies have shown that LLM-based evaluators can approximate human preference judgments in pairwise comparisons, such evaluations may implicitly favor fluency, coherence, and stylistic completeness over domain-specific rigor or evidentiary nuance. Consequently, the comparative preference for responses generated by general-purpose models such as Claude Sonnet 4 should be interpreted as reflecting perceived answer quality rather than definitive scientific correctness. Third, architectural differences among the evaluated systems constrain direct comparison. INSIGHT operates as a closed-domain retrieval-augmented system restricted to a curated document corpus, whereas Perplexity, Claude, and ChatGPT rely on broad parametric knowledge acquired during large-scale pretraining. These systems, therefore, differ fundamentally in information access, evidence grounding, and response generation strategies. Observed differences in answer quality should thus be interpreted as reflecting trade-offs between grounded retrieval and parametric knowledge synthesis rather than the intrinsic superiority of one approach over another. Finally, the number of evaluation questions limits the statistical resolution of performance comparisons. While the analysis provides informative diagnostic insights into retrieval behavior and answer synthesis, larger question sets spanning additional domains and difficulty levels would be required to robustly characterize performance variability and detect more subtle differences between systems. Future evaluations of INSIGHT should extend beyond document-level retrieval metrics to assess the quality of generated narrative syntheses directly. Several complementary evaluation frameworks warrant consideration. First, the RAGA framework (53) provides automated end-to-end evaluation of RAG pipelines through four component metrics: faithfulness (whether generated claims are grounded in retrieved documents), answer relevancy (whether the response addresses the query), context precision (whether retrieved chunks are pertinent), and context recall (whether all necessary evidence was retrieved). Applying RAGA would allow decomposition of overall system performance into retrieval and generation subcomponents, enabling targeted optimization of each pipeline stage. It should be noted that RAGA were not applied in the current study because they require ground-truth answer pairs rather than document-level relevance labels; the evaluation design would need to be modified accordingly in future work. Second, structured comparison between INSIGHT-generated syntheses and published systematic reviews represents perhaps the most rigorous available benchmark for synthesis quality. For each candidate systematic review, INSIGHT would generate a synthesis using only literature available prior to the review’s publication date, and the output would be evaluated against the published review text using STS scoring alongside RAGA metrics, providing a principled and reproducible measure of how closely AI-generated synthesis approximates expert human evidence appraisal. This approach is identified as the primary future evaluation direction in the *Opportunities* section. Third, expert validation by domain specialists in animal science and human nutrition would provide the most direct assessment of synthesis accuracy and scientific rigor, capturing dimensions of domain-specific correctness and interpretive nuance that automated metrics cannot fully represent. Fourth, hallucination detection through citation cross-referencing and semantic consistency scoring, comparing generated claims against the specific passages cited as supporting evidence, would allow systematic quantification of attributability failures, complementing the self-verification step (Stage 9) already implemented in the INSIGHT pipeline. Together, these evaluation approaches would provide a substantially more complete characterization of INSIGHT’s capabilities and limitations as a scientific synthesis tool.

## Conclusion

This study introduced INSIGHT, a domain-specialized retrieval-augmented generation system designed to integrate evidence across animal production and human nutrition research through transparent, document-grounded synthesis. Evaluated across 26 semantically distinct literature groups comprising 282 documents and 614 queries, INSIGHT demonstrated reliable performance (precision = 0.77, recall = 0.62, F1 = 0.67), with all metrics significantly exceeding baseline thresholds (*p* < 0.0001). It is important to emphasize that these metrics reflect document-level retrieval performance, the system’s ability to identify and rank relevant papers from the corpus, and should not be interpreted as measures of generated text accuracy or synthesis quality, which require distinct evaluation frameworks such as Ragas and systematic review benchmarking as described in the Limitations section. Critically, significant between-group variation (*p* < 0.0001) revealed that retrieval effectiveness is context-dependent, with performance differing substantially across topical domains, a finding consistent with emerging evidence that even frontier general-purpose language models exhibit systematic classification drift across nutrition tasks when prompt structure or evaluation criteria change.

These results establish the feasibility of domain-specialized, evidence-grounded language models for cross-disciplinary knowledge synthesis in nutrition science. While further development is needed to improve retrieval coverage, reduce semantic performance gaps across domains, and enhance global terminology normalization, INSIGHT provides a critical foundation for accelerating translation of research findings into actionable guidance. By maintaining explicit provenance and transparent evidence grounding, such systems complement rather than replace expert judgment, supporting evidence-based decision-making while ensuring that continued scientific investment translates into meaningful improvements in food system sustainability and human health outcomes.

## Data Availability

The original contributions presented in the study are included in the article/Supplementary material, further inquiries can be directed to the corresponding author.

## Footnotes

1
https://www.nutritionmodels.com/insight

2
https://fdc.nal.usda.gov/Foundation_Foods_Documentation

3
https://www.dietaryguidelines.gov

4
https://ollama.com/
