# Supplementary material for: Toward an integrated animal–human nutrition intelligence via agentic retrieval-augmented language models
Source: Front Nutr. 2026 Sep 10;13:1815038. doi: 10.3389/fnut.2026.1815038 (PMC13600821; doi:10.3389/fnut.2026.1815038)
Supplement: Supplementary file 1 [file Image_1.PDF]

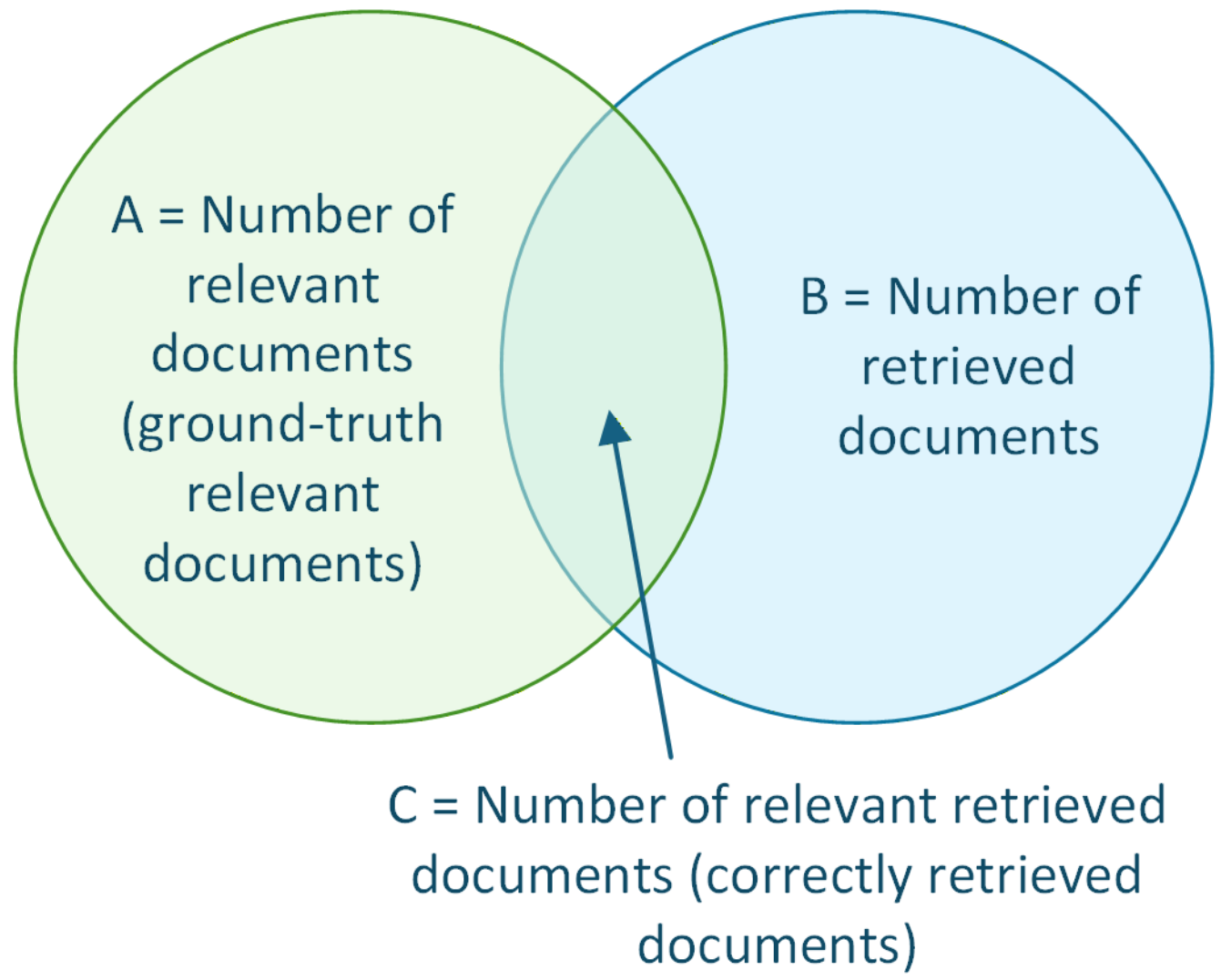

Evaluation metrics: Precision (P) = C/B, Recall (R) = C/A, and F1 score (F1) = 2·P·R/(P + R)

Figure S1. Per-question document counts and evaluation metrics for the INSIGHT system. For each of the 25 questions, the figure shows the number of ground-truth relevant documents, the number of documents retrieved by the INSIGHT RAG system, and the number of relevant documents retrieved, together with the resulting precision, recall, and F1-score.
